# Integration of transcriptomic data in a genome-scale metabolic model to investigate the link between obesity and breast cancer

**DOI:** 10.1186/s12859-019-2685-9

**Published:** 2019-04-18

**Authors:** Ilaria Granata, Enrico Troiano, Mara Sangiovanni, Mario Rosario Guarracino

**Affiliations:** 1 High Performance Computing and Networking Institute, National Research Council of Italy, Via P. Castellino, 111, Napoli, 80131 Italy; 2 Stazione Zoologica Anton Dohrn, Villa Comunale, Napoli, 80121 Italy

**Keywords:** Genome-scale metabolic model, Systems biology, Data integration

## Abstract

**Background:**

Obesity is a complex disorder associated with an increased risk of developing several comorbid chronic diseases, including postmenopausal breast cancer. Although many studies have investigated this issue, the link between body weight and either risk or poor outcome of breast cancer is still to characterize. Systems biology approaches, based on the integration of multiscale models and data from a wide variety of sources, are particularly suitable for investigating the underlying molecular mechanisms of complex diseases. In this scenario, GEnome-scale metabolic Models (GEMs) are a valuable tool, since they represent the metabolic structure of cells and provide a functional scaffold for simulating and quantifying metabolic fluxes in living organisms through constraint-based mathematical methods. The integration of omics data into the structural information described by GEMs allows to build more accurate descriptions of metabolic states.

**Results:**

In this work, we exploited gene expression data of postmenopausal breast cancer obese and lean patients to simulate a curated GEM of the human adipocyte, available in the Human Metabolic Atlas database. To this aim, we used a published algorithm which exploits a data-driven approach to overcome the limitation of defining a single objective function to simulate the model. The flux solutions were used to build condition-specific graphs to visualise and investigate the reaction networks and their properties. In particular, we performed a network topology differential analysis to search for pattern differences and identify the principal reactions associated with significant changes across the two conditions under study.

**Conclusions:**

Metabolic network models represent an important source to study the metabolic phenotype of an organism in different conditions. Here we demonstrate the importance of exploiting Next Generation Sequencing data to perform condition-specific GEM analyses. In particular, we show that the qualitative and quantitative assessment of metabolic fluxes modulated by gene expression data provides a valuable method for investigating the mechanisms associated with the phenotype under study, and can foster our interpretation of biological phenomena.

**Electronic supplementary material:**

The online version of this article (10.1186/s12859-019-2685-9) contains supplementary material, which is available to authorized users.

## Background

A vast majority of diseases is classified as complex, implying that environmental and lifestyle factors, alongside with genetics, might play a crucial role in the onset and progression of the disease itself. Complex diseases infrequently follow the Mendelian laws of monogenic inheritance. Instead, they are caused by a combination of multiple genetic and environmental components, with low heritability [1, 2]. The identification and characterisation of these contributing factors still represent a challenge for researchers, who are compelled to look at the biological phenomenon under study from different perspectives. The traditional analyses that have been used to identify genes responsible for Mendelian traits have not been equally successful in identifying genes and mechanisms underlying complex diseases [3]. In 2000, the World Health Organization has defined obesity as “a complex and incompletely understood disease” and “a key risk factor in the natural history of other chronic and noncommunicable diseases (NCDs)” [4]. In particular, recent evidence highlighted the role of body weight in the development of post-menopausal breast cancer (BC) and the outcome of both posts- and pre-menopausal BC [5–8]. Although several hormonal and metabolic pathways have been investigated to understand the effects of obesity on BC, this connection has not been well characterised so far, and oncologic therapy programs rarely involve weight and lifestyle control. Both the rise of omics sciences and the constant development of the related technologies fostered the research of new approaches based on the integration of data coming from different sources, with the aim to investigate the relationships and the interplay among the various biological molecules [9]. Omics data, made available by high throughput technologies, leveraged the systems biology holistic approach, where the genes are considered as players of complex networks, through which they act and interact [10], and networks are a representation of biological systems, studied as a whole. Investigating a single data type, such as gene expression, DNA variation, metabolic or protein interactions, may lead to incomplete information, while their integration increases the reliability of the results and improves the interpretation of biological phenomena. More specifically, systems biology approaches permit to simulate and describe, through computational and mathematical models, the biochemical transformations occurring into cells and living organisms [11, 12]. Among all biological networks, metabolic networks are probably the best studied, since they directly influence all physiological processes [13]. Indeed, cellular perturbations, determined by genetic and environmental factors, are often driven by and managed through changes in the cell metabolism [14]. In this scenario, GEnome-scale metabolic Models (GEMs) have become a valuable tool for describing and simulating a phenomenon through the definition of a specific set of objects and boundaries, namely a system. A GEM is the representation of the metabolic structure of a cell regarding chemical reactions, involved metabolites, and associated genes [15]. A metabolic network of n metabolites and m reactions can be represented by a stoichiometric matrix, denoted by N, where the entry *N*_*ij*_ represents the stoichiometric coefficient of metabolite i in reaction j [16]. GEMs provide a functional scaffold for constraint-based modeling (CBM) methods aimed at simulating metabolic fluxes in living organisms. Briefly, CBMs interpret a metabolic network as a flow network, whose representation through the stoichiometric matrix is used to compute a solution space, limited by three primary constraints: reaction substrate and enzyme availability, mass and charge conservation, and thermodynamics. Other bounds, derived by specific knowledge of the system, may be used to reduce the size of the solution space. Among CBM methods, Flux Balance Analysis (FBA) is the most used one. It is based on the assumption that an organism aims to maximise a specific cellular metabolic process, recognised as an objective function [17–20]. Usually, in metabolic models of microorganisms, the objective function is the biomass maximisation. Through its optimisation, FBA can identify a single optimal flux distribution that lies on the edge of the allowable solution space. Since the reconstruction of the first global GEM for humans, Recon 1, in 2007 [21], researchers have started to explore the possibility of clinical applications of GEMs [22–29]. The increasing availability of high throughput data is fostering the research of new approaches in which the structural information described by GEMs represent a scaffold for the integration of omics data, with the aim to build condition-specific metabolic states. In particular, omics data can be quantitatively integrated as constraints on the metabolic fluxes to reduce the search space of steady-state solutions [30]. Here we chose a different approach, proposed by Lee et al. [31], which uses a data-driven objective, where the omics data guide the intracellular metabolic fluxes through repeated cycles of their correlation maximisation. The workflow described in our study integrates gene expression data of Luminal-A BC lean and obese subjects into a published reconstructed GEM of the human adipocyte, with the aim to generate condition-specific networks in which gene abundance regulates the metabolic fluxes.

## Materials and methods

### Genome-scale metabolic model and gene expression data

The Genome-scale metabolic model of the human adipocyte, “iAdipocytes1809”, reconstructed and curated by Adil Mardinoglu et al. [26], was downloaded from the Human Metabolic Atlas database in the compressed Systems Biology Markup Language (SBML) format [32]. It represents a functional GEM of the human adipocyte metabolism, generated by immunohistochemistry data of proteins encoded by 14.077 genes of different adipose tissues, along with information from previously published adipocyte-specific proteome data. The model has been validated for 250 known metabolic functions of the adipocytes. Gene expression data were extracted from the dataset GSE78958 [33] deposited at the Gene Expression Omnibus portal. CEL files of 145 Luminal A (Lum A) samples, containing data from 68 lean (BMI < 25) and 77 obese (BMI > 30) patients, were downloaded. Lum A tumour was chosen since is the only subtype for which the authors of the dataset reported significant differentially expressed genes related to body weight. Moreover, to compare the approach used in this study with the FBA analysis performed by the “iAdipocytes1809” developers, the raw files of the dataset GSE27916, from which they extracted differentially expressed genes to incorporate into the GEM, were also downloaded.

### Differential expression analysis

Raw data, in CEL format, were imported, corrected, transformed and normalized through “GEOquery” [34] and “Affy” [35] R packages. The probe ids were converted into the respective gene symbols using the specific annotation file “hgu133a2.db” for GSE78958 and “hgu133plus2.db” for GSE27916. Both the datasets were split into two groups based on body weight (BMI < 25 = lean and BMI > 30 = obese). Mean and standard deviation (SD) of the expression values of all the samples belonging to the same group were calculated. The two resulting files, one for the lean and one for the obese group, containing mean and standard deviation for each gene symbol, were used as input for the model simulation. The differential expression analysis between lean and obese Lum A samples of the GSE78958 dataset was performed using “Limma” R package [36]. The False Discovery Rate approach by Benjamini and Hochberg [37] was applied for multiple-testing adjustment.

### Data integration and model simulation

The algorithm by Lee and colleagues [31], to which we refer as Lee-12, aims at removing the need for an a priori specified objective function, as commonly done in classical FBA approaches. Instead, Lee-12 exploits available expression data to drive the optimisation process by maximising the correlation between the steady-state patterns of the flux solutions and the corresponding gene expression data of the condition under study. The algorithm relies on the COnstraint-Based Reconstruction and Analysis (COBRA) toolbox [38], that was also used to import the “iAdipocytes1809” GEM in SBML format into the Matlab environment. Gene expression mean values and relative standard deviations of lean and obese samples were uploaded individually. To validate our approach we used the transcriptomics dataset reported by the “iAdipocyte1809” authors and compared our results with theirs, obtained by a standard FBA method. The two approaches were compared on the formation of lipid droplets (LDs) as output flux. Clinical fluxomic data of adipose tissue coming from lean and obese subjects, reported by McQuaid et al. [39], have been incorporated into the model to constrain the search space: the lower and upper bound of the fluxes associated with those metabolites were set to the experimentally measured values. In particular, glucose uptake, and triglyceride extraction rates at six different time points were used, being both essentials metabolites for the LDs formation. The reliability of our approach was further evaluated by comparing the rates of non-esterified fatty acids (NEFA) to the clinically measured ones reported by [39]. For the case-study dataset GSE78958, we also set as input values the flux rates of the above cited metabolites at two selected time points, preprandial (tp4) and postprandial (tp5), with the same aim of restricting the searched solution space. The Matlab scripts and the input files are provided in the Additional file 1.

### Graphical model selection

To investigate the topological properties of the metabolic networks, metabolic fluxes obtained by the model simulations were used to build specific graphs and analyse their graph-theoretic measures. For each of the four conditions (lean/obese and preprandial/postprandial uptake), the reactions having zero flux rate were removed, and the directions of the reversible reactions were forced by the sign assigned at the flux rate values during the simulation. Furthermore, recurring metabolites (such as H _2_O, CO _2_, ATP, NADH, etc.) were excluded to avoid an unrealistic definition of the paths [40]. Directed graphs were obtained considering the reactions as nodes, with edges connecting the nodes when shared metabolites were present as a reagent in one reaction and as a product in the other one. The direction was considered from product to reagent. Graphs were built through in-house R scripts. The scripts and the input files are available in the Additional file 2.

Gene-based graphs were built using genes associated with model reactions. Genes were used in place of the reactions they regulate, and the rules underlying the graph building were the same described above for the reaction-based metabolic networks. Two genes were considered connected if they regulate two reactions sharing a metabolite, as product and reagent, respectively. Recurring metabolites were likewise removed, and a direction based on the flux was assigned to reversible reactions. The software has been implemented in Matlab. With this approach we focused, from a gene point of view, on the regulation of metabolic fluxes and its putative alteration in the presence of the disorder under study.

### Structural and differential analysis of metabolic networks

The lists of connections were imported into Cytoscape 3.6.0 [41] and analysed in terms of topological properties. The Cytoscape application Dynet [42] was used to perform a pairwise comparison of the lean and obese networks based on nodes and edges presence, as well as on the ‘rewired’ nodes, identified as nodes different in terms of the identity of the interacting neighbours. DyNet builds a central reference network from the union of the single networks and allows pair-wise comparisons based on selected network attributes.

## Results and discussion

### Approach validation

**Fig. 1:**
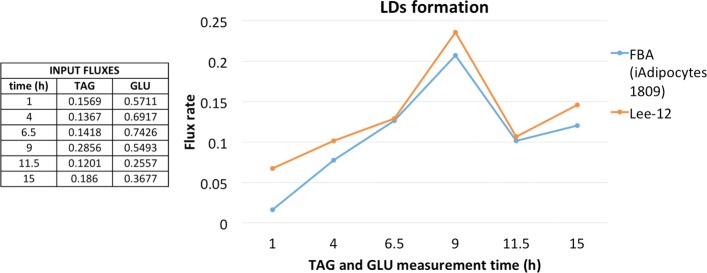
Comparison of lipid droplet flux rates. Comparison of LD fluxes obtained by FBA (iAdipocytes1809) and Lee-12 using six different time points of TAG and GLU fluxes as input. The values are expressed in *μ*mol 100g^−1^ min^−1^

Being Lee-12 originally developed to simulate a *Saccharomyces cerevisiae*’s model, as a first step we decided to compare Lee-12 results with the ones from Mardinoglu et al. [26], in which FBA was uses to estimate the formation of LDs in lean and obese subjects. The flux corresponding to LDs formation was obtained by incorporating the clinical flux rates of specific metabolites, measured in subcutaneous tissue over a 24 h period [39]. To compare the two methodologies, we fed to Lee-12 the gene expression values taken from the same transcriptome dataset (GSE27916) used as reference in [26]. Furthermore, triacylglycerols extraction (TAG) and glucose (GLU) fluxes clinically measured in soft adipose tissue (SAT) of obese subjects at six different time points were used to set the lower and upper bounds of the corresponding metabolites of the model. As previously explained, Lee-12 does not specify an objective function. Since in [26] the defined objective was LDs production, we evaluated the agreement of the two approaches on this flux. The time points selected were: the beginning and the end of the measurement period (time = 1, time = 15), before and after lunch (time = 4 and time = 6.5), before and after dinner (time = 9 and time = 11.5). We used the same input flux values as in [26]. As shown in Fig. 1 the rate of LDs production by Lee-12 and FBA were comparable at all the time points investigated.

**Fig. 2:**
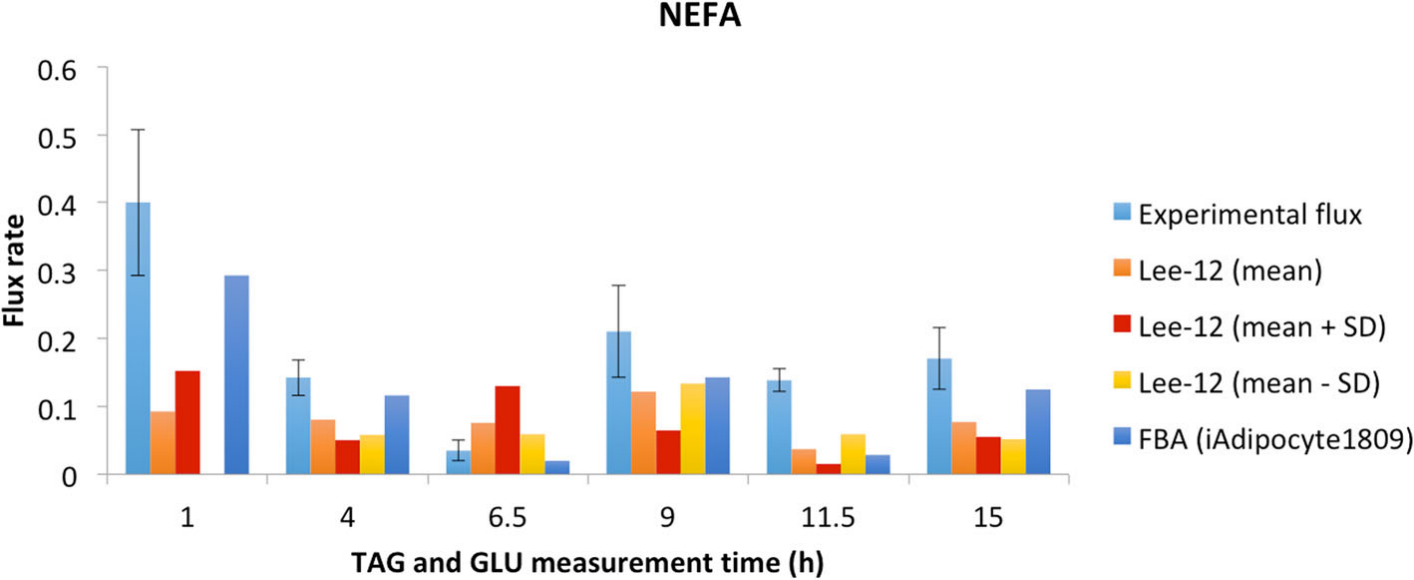
NEFA release rate comparison. NEFA experimental release rate [39] (both mean and SD are reported) and NEFA flux rates by Lee-12 and by reference [26]. We performed three simulations, using the mean, mean plus standard deviation (+SD) and mean minus standard deviation (-SD) of GLU and TAG input fluxes, and the three output values of NEFA fluxes are shown for each time point. The values are expressed in *μ*mol 100g^−1^ min^−1^

The reliability of our approach was further investigated by comparing NEFA release rates to the ones experimentally measured in [39] and used as a reference in [26]. Since these values are reported as mean ± SD, and in most of the cases the latter has very high values, three simulations were performed, using the mean, mean minus SD and mean plus SD of GLU and TAG input fluxes. Output NEFA release rates for all the three simulations are reported in Fig. 2. The values are not always comparable to the experimental ones, nonetheless given the high variability of measurements, shown by their SD, and the integration of gene expression data from an experiment without time points, we can consider the results acceptable. The flux rates have also been compared to the results obtained by the reference [26], in which the authors have added/subtracted the SD to/from the experimental fluxes.

### Differentially expressed genes between lean and obese cancer patients

The differential expression analysis was carried out on microarray data from GSE78958 between lean (BMI < 25) and obese (BMI > 30) subjects affected by Lum A breast cancer. Five genes resulted significantly differentially expressed (Table 1) with four up-regulated and one down-regulated in obese versus lean patients. Our results are partially in agreement with those of [33], most probably due to the *p*-value correction and adjustment they performed on age and ethnicity, factors that we did not take into account in our analysis. APOD and OGN were reported by the authors of the dataset as the mostly up-regulated genes in obese. OGN, which is an osteocyte gene, has also been found to be over-expressed in TNBC (Triple Negative Breast Cancer) subtype [43], and GO annotations related to this gene include growth factor activity. APOD is a well-known gene involved in glucose and lipid metabolic processes, and it has been associated with both obesity and breast cancer [44–46]. A role for ADH1B and its polymorphisms in obesity and insulin resistance has been demonstrated by several works [47]. DLX2 gene has been identified as involved in metastasis risk and aggressiveness of breast cancer [48, 49]. The only down-regulated gene was SYT1, which is involved in synaptic vesicles traffic, a function not rarely associated with obesity [50, 51]. Although few genes have been found differentially expressed according to body weight, the functions in which they are involved, and the strong association with both the diseases under study represented a good starting point to deepen the investigation on their dysregulation effects.

**Table 1:** Differentially expressed genes in obese compared to lean cancer patients

Probe id	Gene symbol	logFC	adj.*p*-value
218730_s_at	OGN	1.12	0.003
207147_at	DLX2	1.21	0.012
201525_at	APOD	1.32	0.023
209613_s_at	ADH1B	1.10	0.025
203999_at	SYT1	-1.14	0.042

Probes having log _2_FC ≥ ∣1∣ and Benjamini-Hochberg FDR corrected *p*-value ≤ 0.05 were considered significant

### Flux rates differences between lean and obese cancer patients

The output flux rate values from the four performed simulations were analysed. In particular, reversible reactions having opposite directions, in lean and obese groups, were identified. Most of them, at both time points, were associated with transport reactions driven by SLC (Solute Carrier) gene family (Additional file 3). SLC are transport proteins located in cell membrane that play a fundamental role in cellular homoeostasis maintenance, and their role in diseases has a great interest for developing new drug targets [52]. The influence of diet on membrane lipid composition is well-known [53, 54], as well as the tight link between the lipid bilayer and the cell functions regulation [55, 56]. The opposite direction of solute transport fluxes suggests a different regulation, in terms of storage and exchange reactions, in obese adipocytes. Furthermore, the ratio of flux rates between obese and lean patient highlighted a generally slower metabolism in obese, with 415 and 382 reactions having lower values than lean (≤ 2FC), at tp4 and tp5, respectively, and only 12 and 37 reactions with higher rates (≥ 2FC) (Additional file 3).

### Metabolic network differences between lean and obese cancer patients

**Fig. 3:**
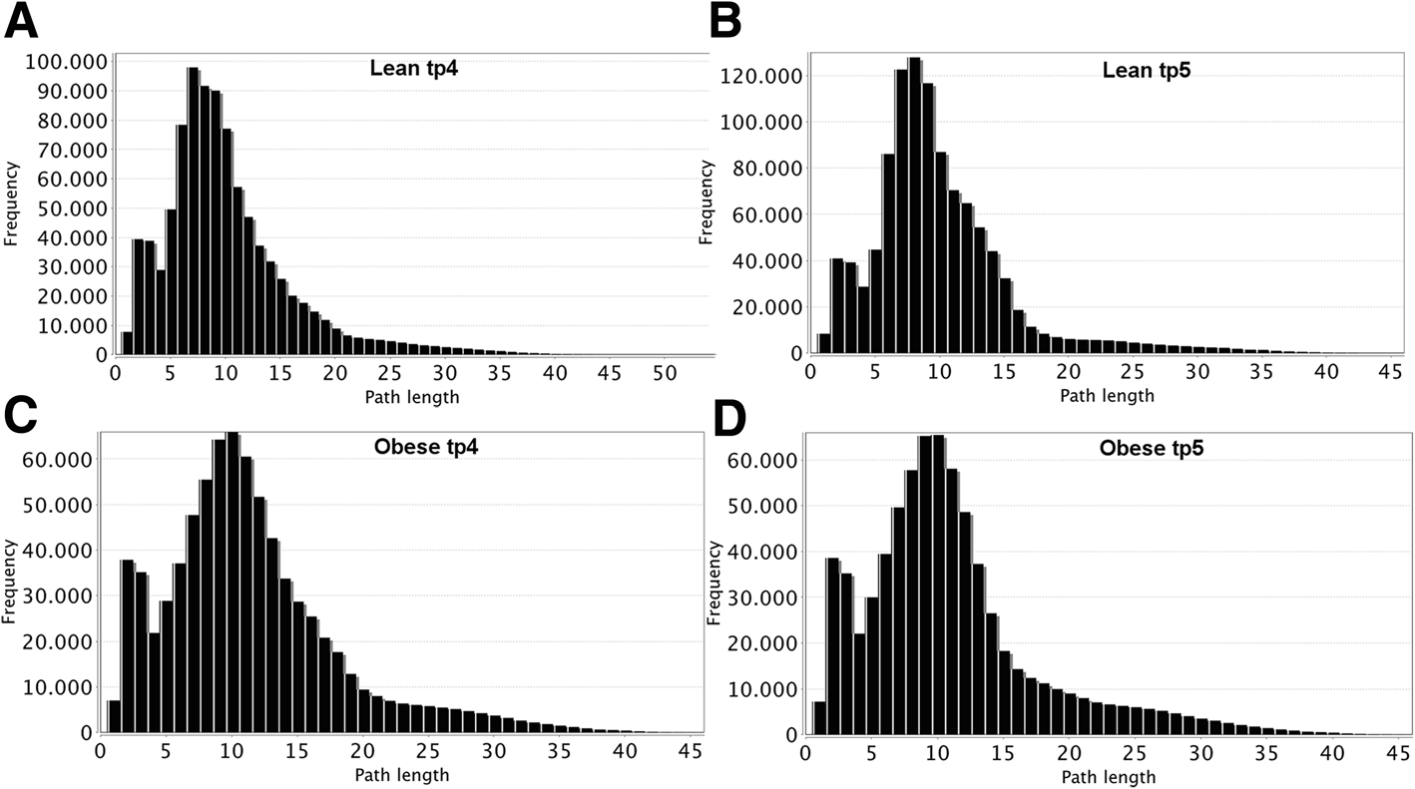
Shortest path distributions. Distribution of shortest paths shows that in lean networks, at both time points (**a**-**b**), path lengths of 7 and 8 nodes have the highest frequency, respectively. Instead, in case of obese networks (**c**-**d**), the most predominant length is 10 steps, indicating longer paths to connect two given nodes

Four different networks were created from the output fluxes obtained by our approach. Specific networks (i.e., graphs) of lean and obese using preprandial and postprandial experimental fluxes were analysed. The connections (i.e., edges) among the reactions (i.e., nodes) were defined by the presence of shared metabolites, with the direction going from the reaction producing a metabolite to the one consuming it. The obtained directed graphs were then imported into the Cytoscape environment, and their topological properties were analysed. Figure 3 shows the distributions of the shortest paths length for the four networks. In lean networks, more reactions and connections are present at both time points, and the highest frequency is at 7 and 8 steps length at preprandial and postprandial time points, respectively. In the case of the obese network, 10 is the most frequent length, suggesting that more steps are needed to connect two nodes and, as a consequence, to complete a metabolic function.

**Fig. 4:**
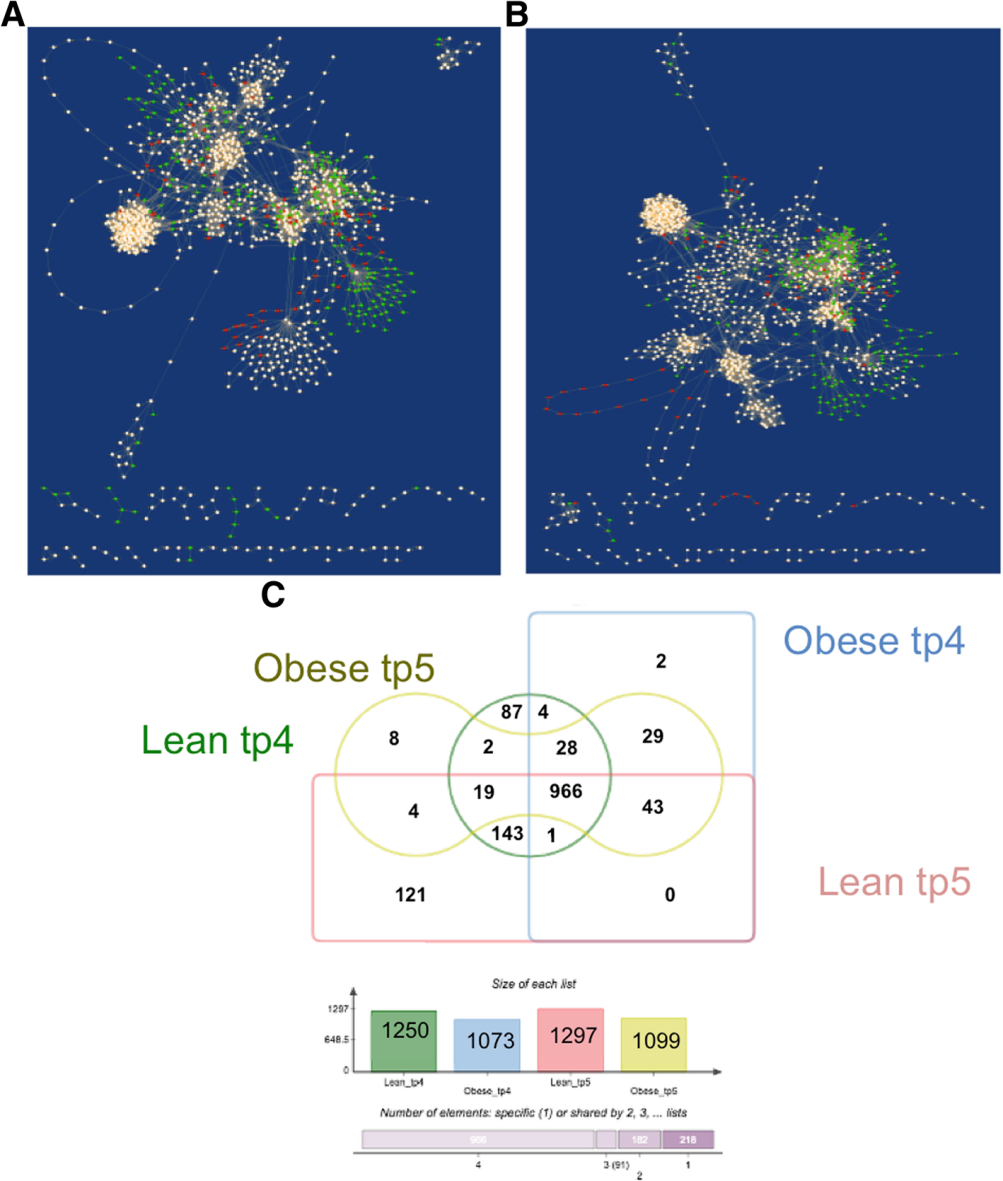
Networks comparison based on nodes presence. Central reference networks were built by DyNet merging lean and obese networks of tp4 (**a**) and tp5 (**b**) fluxes. Common nodes are white, lean-specific nodes are coloured in green, and obese-specific nodes are displayed in red. A prevalence of green and white nodes is present. Intersecting the nodes of each condition, the number of common and specific reactions has been calculated (**c**). A higher number of active reactions is present in lean networks compared to obese ones, and also have much more differences between preprandial and postprandial phase

**Fig. 5:**
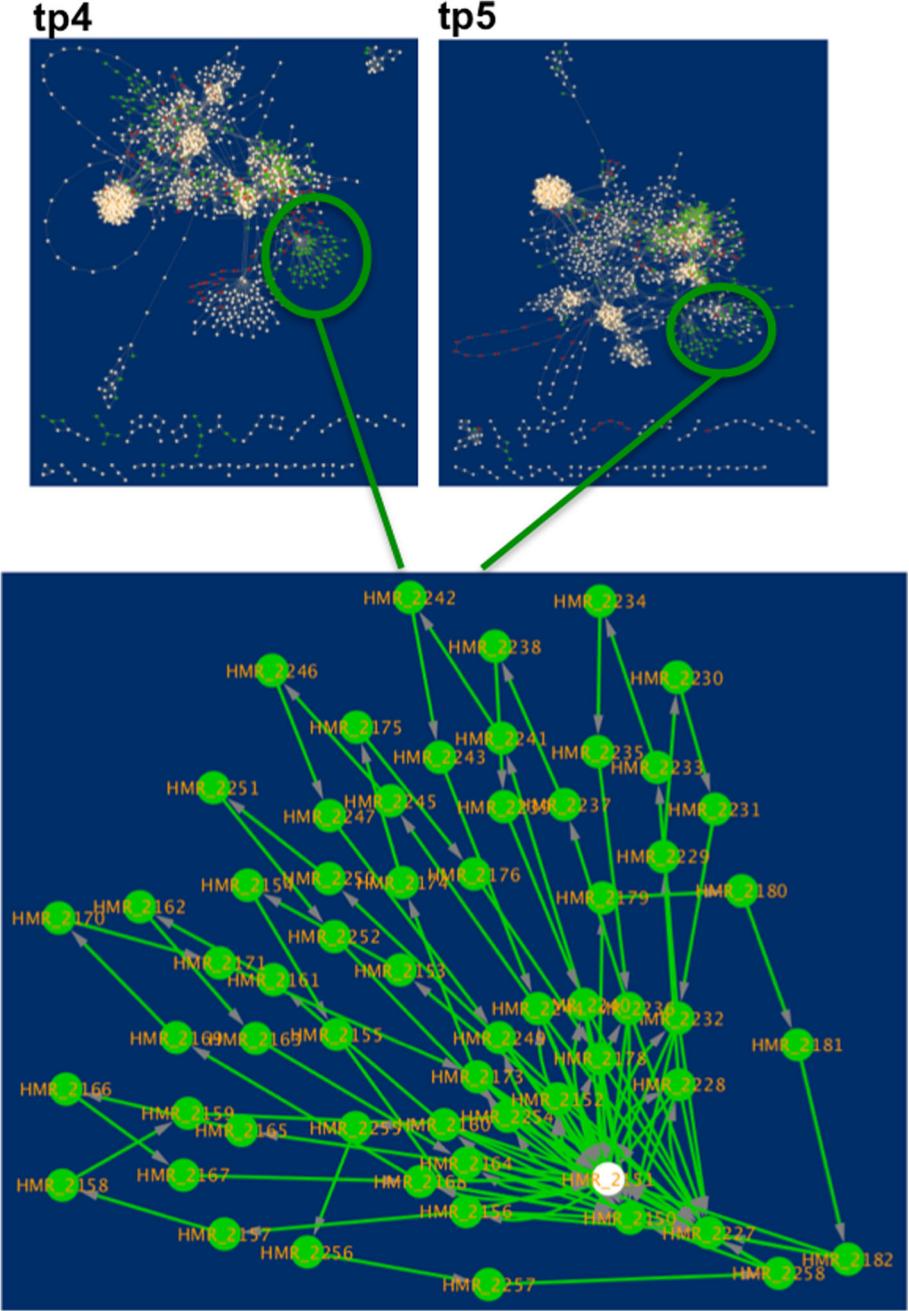
Acyl carrier protein (ACP)-related reactions. Among the lean-specific nodes, it is worth noting a subgroup of reactions involving the acyl carrier protein. As shown in the zoom-in of the central reference networks at tp4 and tp5, all the nodes are green except one. This finding suggests a dysregulation of lipid biosynthesis and metabolism pathways

**Fig. 6:**
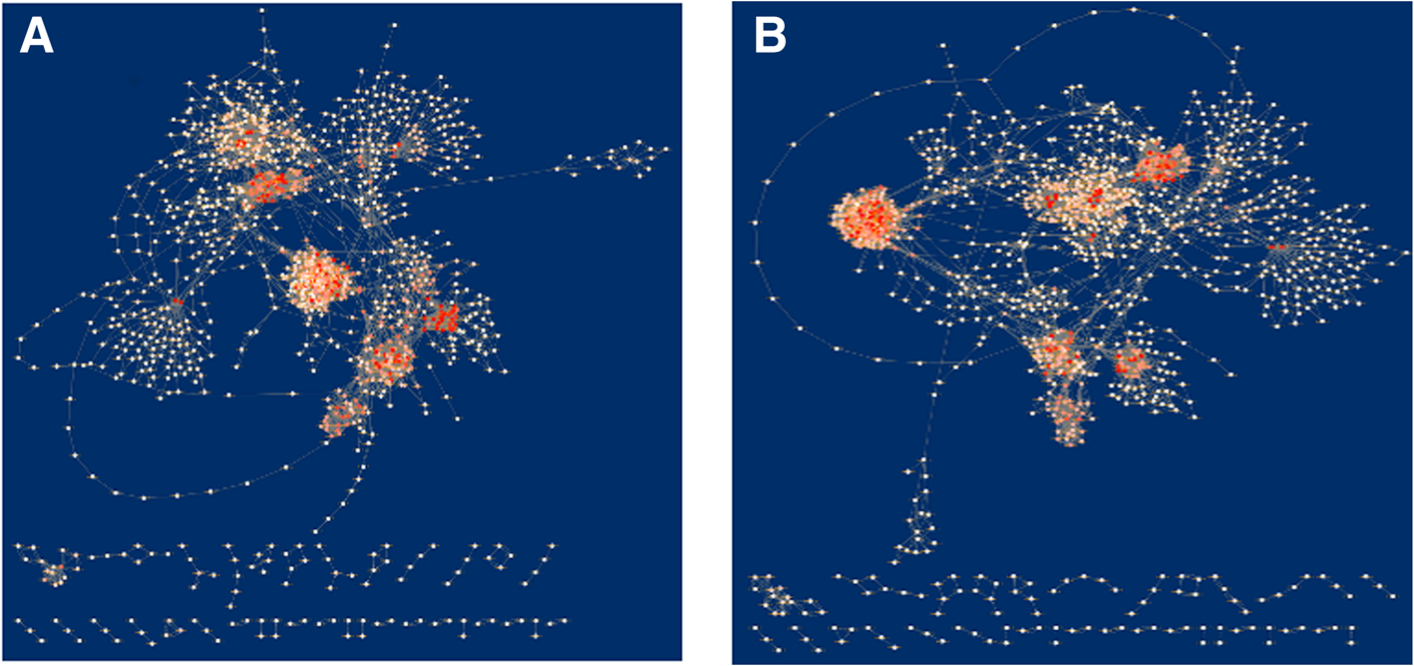
Networks comparison based on rewired nodes. Central reference networks were built by DyNet merging lean and obese networks of tp4 (**a**) and tp5 (**b**) fluxes. Most rewired nodes are highlighted with shades of red, with a more intense colour indicating a higher variation

To perform network comparison and visualise the results, the Cytoscape application DyNet was used. Comparison of networks in terms of nodes presence for each time point highlighted a high number of nodes specific to the lean condition which are repressed in obese networks (Fig. 4a-b). In particular, lean networks contain 177 and 198 more reactions than obese at tp4 and tp5 respectively (Fig. 4c). Furthermore, it is worth to notice that lean networks show major changes when the two time points are compared, while obese networks have a very low percentage of different nodes between the preprandial and postprandial phase. Indeed, lean network at tp5 contains 121 unique reactions. This evidence suggests that the food intake influences much more a working metabolism compared to the one affected by a metabolic disorder. Among the reactions specific to the lean condition, there is an evident group of 61 nodes (Fig. 5) involving the acyl carrier protein (ACP). ACP is a key cofactor protein that covalently binds all fatty acyl intermediates via a phosphopantetheine linker during the fatty acid (FA) synthesis process [57]. Down-regulation of lipogenic pathways in obesity has been reported as a defence mechanism to avoid the excessive accumulation of fatty acids [58]. DyNet calculates a score (Dn-score) to highlight the most variable nodes on the central reference network, using a colour gradient (Fig. 6a-b). The Dn-score is a rewiring metric of the nodes, which quantifies the changes in the identity of interacting neighbours. Figures 6a and 6b represent the combined network which shows the most rewired nodes at tp4 and tp5, respectively.

The nodes were ranked based on the Dn-score, and the top twenty were further investigated. At both time points, almost all these reactions involved the amino acid transport (Additional file 4), an effect already emerged from the analysis of the opposite direction reactions. A strong link between amino acid transporters and cancer has been investigated by several studies, which highlighted the importance of amino acid availability to support cancer “metabolome” [59–62].

### Gene-based networks differences between lean and obese cancer patients

The comparison of lean and obese networks was performed also considering, in place of the reactions, the associated genes as nodes with the aim to: i) investigate the relationships among genes associated with metabolic reactions; ii) identify the key regulators of the cellular functions involved, and their putative alteration in obese cancer patients. The top 50 rewired nodes from the previous networks comparison were extracted, and the associated genes were enriched in terms of Gene Ontology Biological Processes (Additional file 5) by the Database for Annotation, Visualization and Integrated Discovery (DAVID) 6.8 [63]. From the enrichment analysis, terms as “fatty acid metabolism” and “amino acid transport” confirmed the dysregulation hypothesis stated above. The response to insulin is enriched in the postprandial phase. Although the overall situation does not change between the two time points in obese condition, this result highlights the importance of considering and knowing the sampling time, especially when metabolic disorder are under investigation. Moreover, terms related to oxidative stress and glutathione metabolism were significantly enriched, suggesting interesting insights to understand the relationship between obesity and cancer. Indeed, both of these mechanisms are reported in the literature as associated with cancer onset and progression. In particular, glutathione participates in several functions, such as cell differentiation, proliferation, and apoptosis, and the alteration of its regulation is linked to many human diseases, including cancer [64]. Glutathione has also been associated with oxidative stress [65], a mechanism determined by the accumulation of Reactive oxygen species (ROS) inside the cells, and closely related to inflammation, ageing and cancer [66]. It is well known how these three phenomena have in common a lot of characteristics, as multiple faces of the same event. Since the obesity has started to be considered a disease, it has been associated with immune and inflammatory manifestations [67].

## Conclusions

Here we propose a systematic approach to study complex diseases based on the integration of gene expression data into genome-scale metabolic models. As a case study, we investigated the relationship between obesity and cancer. Although obesity has been associated with a higher risk of developing breast cancer in postmenopausal women, and with worse outcome for women of all ages, further studies are needed to define the biological mechanisms behind. To this extent, starting from a metabolic model of the human adipocyte we integrated experimental data of lean and obese Lum A BC patients using a published algorithm that does not explicitly defines an objective function like otherwise done in FBA. The algorithm we used was originally designed for the analysis of *Saccaromyces cerevisiae* metabolism, and we applied it for the first time on the GEM of a complex organism. From our analyses, several biological inferences have been drawn. Some of them support the validity of the method, confirming well-known mechanisms associated with obesity disorder, such as a lower metabolic rate [68], and the alteration of fatty acid related reactions [69]. Through a topological comparison of the constructed networks, top dysregulated reactions have been identified. In particular, it is worth noting the involvement of amino acid transport, which is a recent issue associated with cancer progression and malignancy [70, 71]. Indeed, since tumour cells have a higher demand for nutrients and molecules to increase their proliferation, it is not surprising that the amino acid related metabolism underlies an alteration of its regulation. Also, obesity is characterized by an increased metabolic demand. Thus, a cumulative effect could determine the worst prognosis of cancer related to body weight. Moreover, the analysis of the networks in terms of both reactions and associated genes allowed to gain insights from different points of view: the study of gene-based networks highlighted a higher variability of the connections related to genes involved in oxidation stress response in the obese group when compared to lean. In the future, additional analyses and validations will be performed, as well as further investigation by including more datasets, and healthy control samples, as well as using other disease models [72, 73]. An exciting future perspective is also represented by the integration of microRNAs (miRNAs) expression data into metabolic models. miRNAs are involved in gene expression and have been associated with obesity, playing an important role in response to changes in environmental conditions, diet and physical activity [74, 75]. Nonetheless, we think this study represents an important proof of concept in the scenario of novel systems biology integrative approaches, which will, in turn, improve our understanding of complex biological phenomena.

## Additional files

Additional file 1 Matlab code and the relative expression input files to simulate the metabolic model by integrating gene expression data. (ZIP 367 kb)

Additional file 2 R scripts and the relative input files to create the reaction-based graphs. (ZIP 368 kb)

Additional file 3 Tables of the flux rates output having either opposite direction or same direction with abs(log _2_FC) ≥ 1 in lean and obese metabolic network. For each table of fluxes, we also provided the table with the expression values (mean and SD) of the associated genes. (XLSX 304 kb)

Additional file 4 Top 20 rewired nodes of tp4 and tp5 central reference networks, obtained by the union and comparison between lean and obese networks. The table contains for each HMR code, the equation, the subsystem and the associated genes. (XLSX 52.9 kb)

Additional file 5 Enrichment analysis of top 50 rewired nodes of tp4 and tp5 central reference gene-based networks, obtained by the union and comparison between lean and obese networks. The list of the rewired genes and the relative Dn-scores is also provided. (XLSX 17.6 kb)

## Abbreviations

ACP: Acyl carrier protein
BC: Breast cancer
BMI: Body mass index
CBM: Constraint-based modeling
FA: Fatty acid
FBA: Flux balance analysis
GEM: GEnome-scale metabolic model
LD: Lipid droplet
NEFA: Non esterified fatty acids
SAT: Soft adipose tissue
SBML: Systems biology markup language
SD: Standard deviation
SLC: SoLute carrier
TAG: Triaglyceride
